# Serum-based metabolic alterations in patients with papillary thyroid carcinoma unveiled by non-targeted 1H-NMR metabolomics approach

**DOI:** 10.22038/IJBMS.2018.30375.7323

**Published:** 2018-11

**Authors:** Reyhaneh Farrokhi Yekta, Mostafa Rezaei Tavirani, Afsaneh Arefi Oskouie, Mohammad Reza Mohajeri-Tehrani, Ahmad Reza Soroush, Alireza Akbarzadeh Baghban

**Affiliations:** 1 Proteomics Research Center, Faculty of Paramedical Sciences, Shahid Beheshti University of Medical Sciences, Tehran, Iran; 2 Department of Basic Sciences, Faculty of Paramedical Sciences, Shahid Beheshti University of Medical Sciences, Tehran, Iran; 3 Endocrinology and Metabolism Research Center, Endocrinology and Metabolism Clinical Sciences Institute, Tehran University of Medical Sciences, Tehran, Iran; 4 Department of Surgery, Shariati Hospital, Tehran University of Medical Sciences, Tehran, Iran; 5 Department of Basic Sciences, School of Rehabilitation, Shahid Beheshti University of Medical Sciences, Tehran, Iran

**Keywords:** Metabolomics, Multinodular goiter, NMR, Serum, Thyroid cancer

## Abstract

**Objective(s):**

As the most prevalent endocrine system malignancy, papillary thyroid carcinoma had a very fast rising incidence in recent years for unknown reasons besides the fact that the current methods in thyroid cancer diagnosis still hold some limitations. Therefore, the aim of this study was to improve the potential molecular markers for diagnosis of benign and malignant thyroid nodules to prevent unnecessary surgeries for benign tumors.

**Materials and Methods:**

In this study, 1H-NMR metabolomics platform was used to seek the discriminating serum metabolites in malignant papillary thyroid carcinoma (PTC) compared to benign multinodular goiter (MNG) and healthy subjects and also to better understand the disease mechanisms using bioinformatics analysis. Multivariate statistical analysis showed that PTC and MNG samples could be successfully discriminated in PCA and OPLS-DA score plots.

**Results:**

Significant metabolites that differentiated malignant and benign thyroid lesions included citrate, acetylcarnitine, glutamine, homoserine, glutathione, kynurenine, nicotinic acid, hippurate, tyrosine, tryptophan, β-alanine, and xanthine. The significant metabolites in the PTC group compared to healthy subjects also included scyllo- and myo-inositol, tryptophan, propionate, lactate, homocysteine, 3-methyl glutaric acid, asparagine, aspartate, choline, and acetamide. The metabolite sets enrichment analysis demonstrated that aspartate metabolism and urea cycle were the most important pathways in papillary thyroid cancer progression.

**Conclusion:**

The study results demonstrated that serum metabolic fingerprinting could serve as a viable method for differentiating various thyroid lesions and for proposing novel potential markers for thyroid cancers. Obviously, further studies are needed for the validation of the results.

## Introduction

Thyroid cancer is the most common malignancy of the endocrine system, head, and neck, which accounts for nearly 4% of total cancer cases in the United States in 2016 (1), however, thyroid nodules are very common with a fast-rising incidence in the last decade (2). Although most of the thyroid nodules are benign and about 5–10% are cancerous (3), identification of thyroid malignancy and development of a suitable treatment for the disease is still a concern for clinicians. Main types of thyroid cancer include papillary thyroid carcinoma (PTC), follicular thyroid carcinoma (FTC), medullary thyroid carcinoma (MTC), and undifferentiated anaplastic thyroid carcinoma (ATC). PTC is derived from follicular thyrocytes which represent more than 80% of all thyroid malignancies (4). The gold standard for diagnosis of thyroid cancers and benign nodules is the fine-needle aspiration biopsy (FNAB) method followed by cytopathological diagnosis (5). However, in about 25% of the cases, the results of FNA are inconclusive which in 80% of the cases with benign nodules would impose an unnecessary surgery (6). Determination of useful biomarkers for the detection of malignancy in thyroid nodules is currently in progress and various molecular markers have been proposed including mutation-based markers like B-RAF, NRAS, PAX8/PPARγ, and RET/PTC translocations (3), and immunocytochemistry based markers like galectin3, fibronectin, thyroglobulin, CD44V6, cytokeratin 19, HBME-1, VEGF, and Aurora-A (7), but the introduced markers for thyroid cancer do not have good specificity and positive predictive value (8). Therefore, determination of reliable molecular markers, especially in biological fluids like urine and blood serum with less invasive collecting methods, is an urgent need as a complement to current methods in order to prevent unnecessary surgeries in benign cases. From omics approaches, metabolomics has a great promise in finding potential markers for various pathological conditions and understanding involved biochemical pathways in disease mechanisms (9). Metabolites serve as the end-products of biochemical reactions in the body, so they are the closest molecules to phenotype. Previous metabolomic studies were based on *in vivo* or *ex vivo* NMR spectroscopy, GC-MS, and LC-MS, which mostly investigated tissue samples where limited work was performed on biological fluids such as urine, serum, or plasma, which can be collected less invasively (10). According to previous studies, the most important metabolites in thyroid tumor tissues included glucose, lactate, citrate, galactose, scyllo- and myo-inositol, hypoxanthine, taurine, inosine, and amino acids especially phenylalanine and glutamate. Tissue levels of lipids, including cholesterol and choline-containing lipids, were also among the significant differences between cancerous and normal tissues. In the current study, we performed a ^1^H-NMR metabolomics approach to find significant relevant metabolites in PTC serum samples compared to multinodular goiter and healthy controls according to the need for less invasive methods and more accessible sources of biomarkers for PTC and MNG. Metabolite sets enrichment analysis and metabolic network analysis was further performed to investigate the involved biochemical pathways in PTC progression.

## Materials and Methods

***Participants and sample collection***

Venous blood in fasting state in the morning was collected in vacutainer tubes and left to clot for 30 min, followed by centrifugation at 3000 g for 10 min at 4 °C. The supernatant serum was aliquoted and stored at -80 °C until required. This study was approved by the local ethics committee for clinical studies of Shahid Beheshti University of Medical Sciences. The patients signed written informed consents. Subjects included 17 patients with PTC, 17 patients with multinodular goiter (MNG) and 20 healthy volunteers all from the same age and gender. All PTC patients were selected from stages I and II of cancer, based on the TNM classification system. The patients were diagnosed with the disease based on both FNA cytology results and histopathologically after thyroidectomy surgery. The patients had no history of carcinomas or diabetes, and they had not started medication at the time of the study. Smokers were also excluded from the study. The patients were from 20 to 60 years old and other ages were discarded. Blood collection lasted for 6 months in 2016 in the surgery Department of Shariati Hospital, Tehran, Iran.

***NMR spectroscopy***

All experiments were performed using a Bruker-Avance 400 MHz, equipped with a 5 mm probe at 298 K. 400 μl of serum samples were mixed with an equal amount of buffer, including 70% D_2_O, 2.5% of TSP (as an internal standard), 4% KH_2_PO_4_, and 0.01% NaN_3_. One-dimensional CPMG (Carr-Purcell-Meiboom-Gill) analysis was the method of choice acquired by a standard pulse sequence, which irradiates residual water peak, with relaxation delay of 2 sec and mixing time of 100 ms. A total of 150 scans were collected over a spectral width of 8389.26 Hz with a 90° pulse width, and the repetition time of 3 sec. 0.3 Hz line broadening was applied to the spectra prior to Fourier transformation and phase- and baseline-correcting. The spectra were referenced to the TSP standard at 0 ppm. The NMR spectra were imported to ProMetab3, which is a software working under MATLAB. The spectra were binned between 0.2 and 10 ppm in 0.01 ppm parts, normalized with the TSA method, aligned to a reference spectrum and log transformed. The spectral region of 4.5-5.5 ppm was also excluded to remove water suppression variation efficiency. The spectra were log transformed and normalized to reduce the effect of the large dynamic range between the concentrations of different metabolites. 

**Figure 1:**
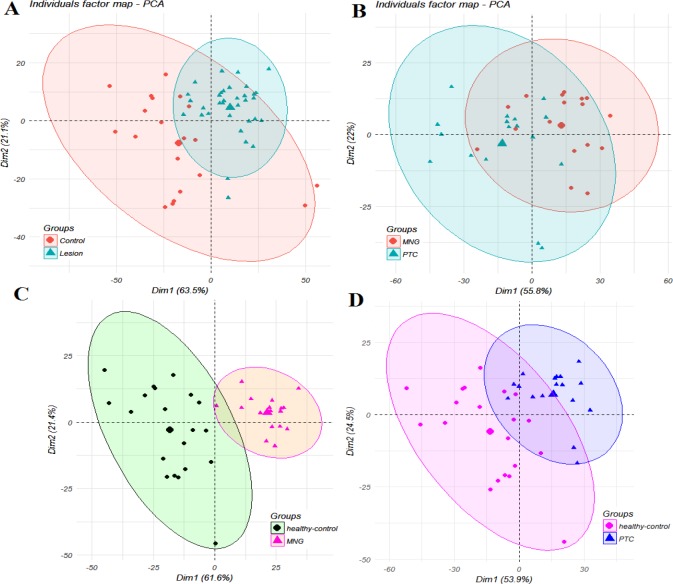
Scatter score plots of the PCA analysis for the discrimination between A) thyroid lesions (PTC+MNG) and healthy controls, B) PTC and MNG, C) MNG from healthy subjects, and D) PTC from healthy subjects

**Figure 2:**
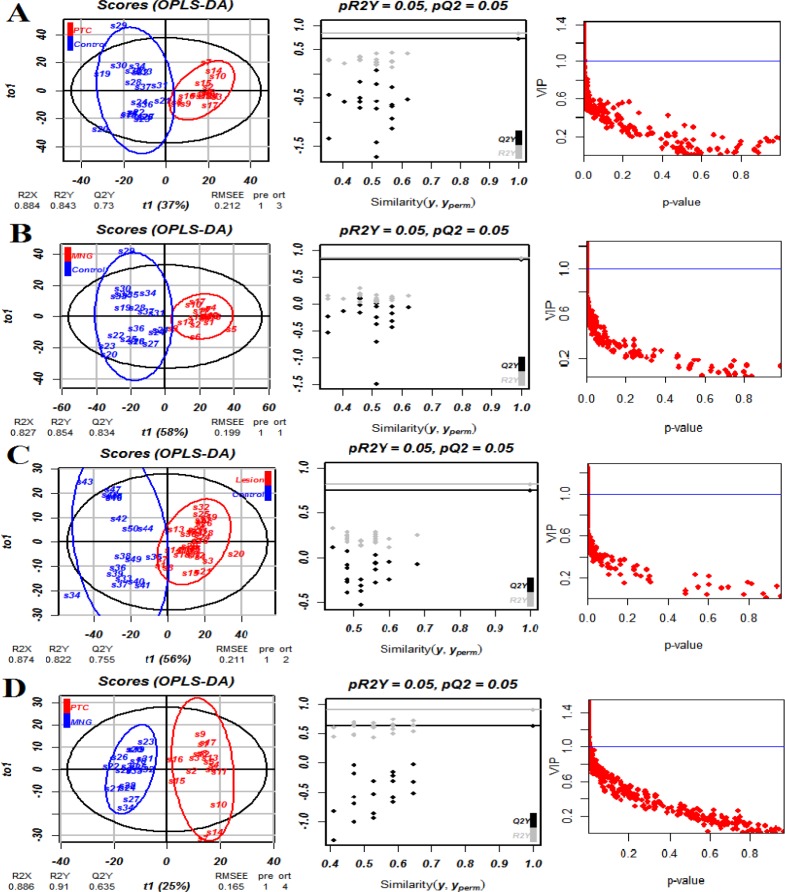
OPLS-DA score plots showing discrimination between A) PTC and healthy controls, B) MNG and healthy controls, C) PTC+MNG and normal subjects, and D) PTC and MNG. As can be seen, OPLS-DA could successfully discriminate the study groups. The results of the permutation testing are shown in the second column. The third column shows the graph of VIP values against the *P*-values

**Figure 3:**
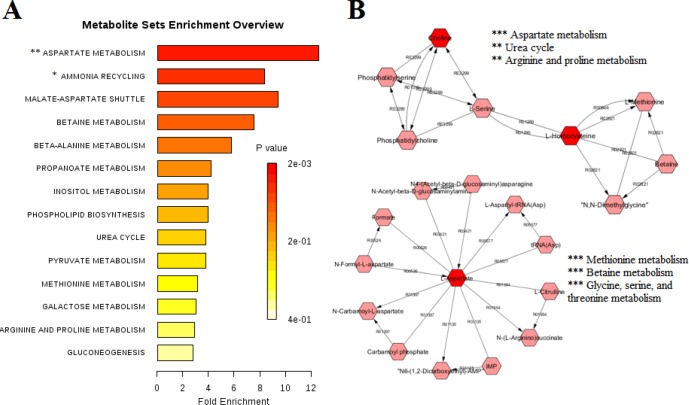
Metabolite sets enrichment analysis (MSEA) results for papillary thyroid carcinoma: A) MSEA for the significant metabolites identified in PTC serum samples and B) MSEA for the modules derived from the PTC metabolic network. (*** *P*<0.001, ** *P*<0.01, * *P*<0.05)

**Figure 4:**
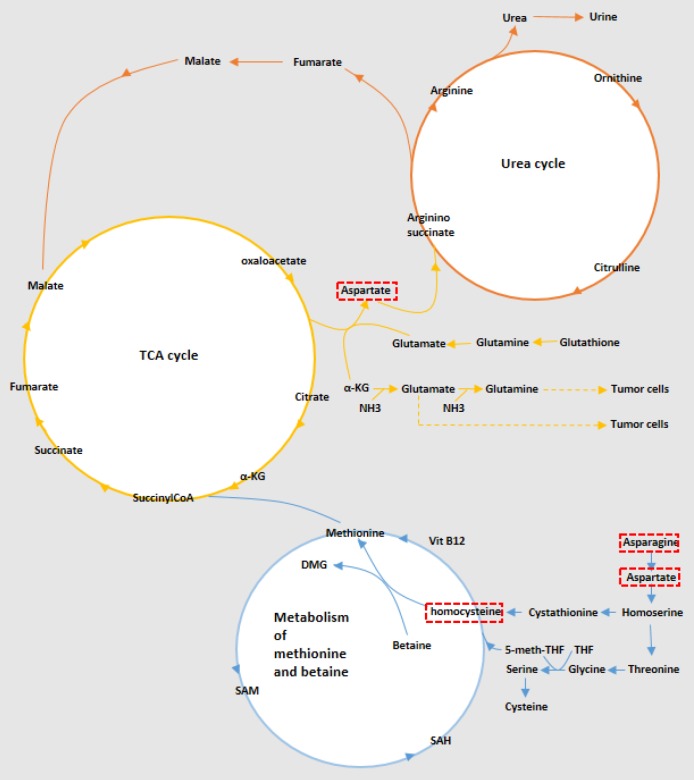
The most significantly enriched pathways involved in the pathogenesis of papillary thyroid carcinoma, including aspartate metabolism, urea cycle, and metabolism of methionine and betaine. The metabolites in the red boxes were significantly altered

**Table 1:** The parameters of the OPLS-DA model and the predictive performance of the model after permutation testing for the training and test sets

	R2X	R2Y	Q2Y	RMSE	Training set		Test set	
					Sensitivity	Specificity	Sensitivity	Specificity
PTC vs Ct	0.884	0.843	0.73	0.212	100%	100%	80%	100%
MNG vs Ct	0.827	0.854	0.834	0.199	100%	100%	100%	100%
PTC vs MNG	0.886	0.91	0.635	0.165	100%	100%	80%	100%
Lesion vs Ct	0.874	0.822	0.755	0.211	93.7%	88.8%	100%	100%

**Table 2:** The significant discriminating serum metabolites between the study groups

Metabolite*	Kegg ID	L *vs* H		PTC *vs* MNG		PTC *vs* H		MNG *vs* H	
		VIP	Fold	VIP	Fold	VIP	Fold	VIP	Fold
Carnitine	C00487							1.20	4.37^a^
Glutaric acid	C00489							1.16	2.64^a^
Succinate	C00042							1.12	2.08^a^
Citrulline	C00327							1.11	1.88^a^
Acetate	C00033							1.11	2.12^a^
Acetone	C00207							1.09	2.15^a^
Alanine	C00041							1.08	1.65^a^
Valine	C00183							1.04	1.51^a^
Glutamine	C00064			1.10	0.66^a^			1.10	1.73^a^
Hypotaurine	C00519	1.20	4.88^a^					1.17	3.48^a^
Citrate	C00158	1.21	5.16^a^	1.24	0.64^a^			1.18	4.41^a^
O-Acetylcarnitine	C02571	1.34	6.10^a^	1.12	0.64^a^			1.15	3.00^a^
GSH	C00051	1.15	2.70^a^	1.06	0.71^a^			1.20	5.16^a^
Methionine	C00073	1.42	2.20^a^					1.13	5.60^a^
Isoleucine	C00407	1.03	1.41^a^					1.06	1.63^a^
Scyllo-inositol	C06153	1.40	2.70^a^			1.01	2.30^a^	1.08	3.16^a^
Tryptophan	C00078	1.48	2.00^a^	1.15	0.40^b^	1.01	2.20^a^	1.19	4.70^a^
Propionate	C00163					1.30	2.20^a^	1.16	2.64^a^
Myo-inositol	C00137					1.01	2.28^a^	1.10	5.00^a^
Lactate	C00186					1.15	1.51^c^	1.03	2.20^a^
Homocysteine	C00155					1.28	2.18^a^		
3-methyl glutaric acid	C03761					1.16	2.00^a^		
Asparagine	C00152					1.12	3.03^c^		
Aspartate	C00049					1.09	2.44^a^		
Acetamide	C06244					1.01	0.25^a^		
Choline	C00114	1.19	4.16^a^			1.06	3.56^a^		
Kynurenine	C01717	1.18	0.45^a^	1.74	1.60^b^				
Hippurate	C01586	1.10	1.91^a^	1.30	2.71^a^				
Tyrosine	C00082	1.18	2.11^a^	1.32	0.55^a^				
Nicotinic acid	C00253			1.40	2.50^a^				
Xanthine	C00385			1.15	3.00^a^				
Homoserine	C00263			1.08	0.70^a^				
β-alanine	C00099			1.21	0.70^b^				

PTC: papillary thyroid carcinoma, MNG: multinodular goiter, VIP: variable importance for the projection.

a : significant with *P*-value<0.001,

b : significant with *P*-value<0.01,

c : significant with *P*-value<0.05,

L : thyroid lesion,

H : healthy control,

* Those metabolites which had VIP values more than 1, fold change more than 1.4 and *P*-values less than 0.05 are considered.

***Statistical and bioinformatics analysis***

The resulting dataset was imported to “R” v.3.3.2 for multivariate statistical analysis. The unsupervised principal component analysis (PCA) was used to identify similarities or trends in different samples. It could also reveal the outliers and relationships that exist between observations. The supervised method, orthogonal projections to latent structures-discriminant analysis (OPLS-DA) was done to maximize the covariance between measured data (x matrix or peak intensities of the NMR spectra), searching for x-variables that correlate with class members (classification) so that the variable could predict the response y. PCA was performed using the Factoextra package. The OPLS-DA was also performed using the ropls package. The significant metabolites were identified using the biological magnetic resonance databank (BMRB) (11) and the human metabolome database (HMDB) (12). Metaboanalyst3 web-based platform was used for pathway enrichment analysis (13). Two-sided student’s t-test was used to evaluate *P*-values for each metabolite. The *P*-values were then Bonferroni-corrected due to the large number of variables. Variable influence on projection (VIP) was used to find the most significant discriminating metabolites in each comparison. Metabolites with VIP>1, *P*-value<0.05, and fold changes of more than 1.4 were marked as significant variables, which are presented in Table 2. Metabolic network was constructed in cytoscape3 using Metscape application (14, 15). 

## Results

Multivariate analysis was performed on the result matrix to find metabolites that mostly discriminated the study groups. Principal component analysis (PCA) was the unsupervised analysis method, which was used for dimension reduction of data through making a linear combination of variables known as principal components. PCA analysis can reveal trends in the data and groups of observations and find outliers. The PCA results are shown in Figure 1. The variation that was explained by the first three components for discriminating both thyroid lesions (PTC+MNG) from healthy controls by the PCA analysis was as follows: PC1=63.5%, PC2=21.1%, and PC3=4.9%. The values for discrimination of malignant and benign classes were 55.8%, 22%, and 6.4%, respectively. We also compared PTC and MNG groups with healthy subjects. The first three components’ values for discriminating PTC and healthy controls were 53.9%, 24.5%, and 10.6%, whereas these values were 61.6%, 21.4%, and 8.8% for the comparison of MNG and healthy subjects. As Figure 1 clearly shows, PCA plots show acceptable discrimination of the study groups. All samples in PCA score plots were within the 95% Hotelling’s T2 ellipsoid except for the discrimination of thyroid lesions (PTC+MNG) and healthy controls where there was an outlier for the control and 2 outliers in the lesion samples. We omitted these nodes for the execution of the OPLS-DA model. OPLS-DA is a supervised analysis method that is employed to divide the samples into different groups, including diseased and healthy, which was performed to find metabolites that mostly discriminated the studied groups in each comparison. The OPLS-DA analysis of the NMR metabolic profiles of malignant, benign and healthy controls could successfully separate clusters for each comparison, which is visualized in score plots in Figure 2. Validation of the OPLS-DA model is typically described by R2 (coefficient of determination) and Q2 (cross-validated R2), which was performed here by permutation testing. The permutation test for the OPLS-DA model resulted in values of R^2^X, R^2^Y, and Q^2^Y of 0.884, 0.843, and 0.73 for discriminating PTC from healthy controls. These values were 0.827, 0.854, and 0.834 for the discrimination of benign MNG from the healthy group, all of which indicated the effectiveness of the models. The RMSE values were 0.212 for PTC-control and 0.199 for MNG-control comparisons. The permutation testing also demonstrated the R^2^X value of 0.87, the R^2^Y value of 0.822, and Q^2^ value of 0.755 for discriminating thyroid lesions from healthy controls with the RMSE value of 0.211. The values of R^2^X, R^2^Y, Q^2^Y, and RMSE were 0.886, 0.91, 0.635 and 0.165 for differentiation of benign from malignant nodules. All models had Q^2^ values greater than 0.4 with the intercept of less than zero, which demonstrated that the models were validated. The OPLS-DA model parameters are demonstrated in Table 1. Variable influence on projection (VIP) from the OPLS-DA models could reveal the significantly altered metabolites. We selected the metabolites with VIP>1 and Bonferroni-corrected *P*-values less than 0.05. The cutoff for fold-change was also set 1.4. The resulted spectral bins were then searched in the BMRB and HMDB databases to find metabolites that matched the magnetic resonance spectral bins. The resulted metabolites are shown in Table 2. According to the results, increased amounts of tryptophan, hypotaurine, citrate, choline, hippurate, methionine, scyllo-inositol, acetyl-carnitine, glutathione, tyrosine, and isoleucine were observed in thyroid lesions (including PTC+MNG) compared to healthy controls where kynurenine level was decreased. The discriminating metabolites between PTC and MNG samples also included the decrement of citrate, acetylcarnitine, glutamine, homoserine, tryptophan, tyrosine, β-alanine, and glutathione along with increased kynurenine, nicotinic acid, hippurate, and xanthine. Twenty metabolites were also selected for the MNG group compared to normal subjects including carnitine, tryptophan, glutathione, citrate, hypotaurine, glutaric acid, propionate, O-acetylcarnitine, methionine, succinate, citrulline, acetate, glutamine, myo-inositol, acetone, alanine, scyllo-inositol, isoleucine, valine, and lactate, which were all increased in MNG serum samples. Altered metabolites for the PTC group compared to healthy control included homocysteine, propionate, 3-methyl glutaric acid, lactate, asparagine, aspartate, choline, acetamide, myo-inositol, scyllo-inositol, and tryptophan. Metabolite sets enrichment analysis for the PTC altered metabolites and metabolic network modules demonstrated that aspartate metabolism, urea cycle, and metabolism of methionine and betaine were the most significant pathways involved in the pathogenesis of papillary thyroid cancer.

## Discussion

The metabolic profile survey is considered an efficient method for the identification of diagnostic biomarkers and also a tool for better understanding of the disease pathology. The metabolomic study of body fluids, especially blood and urine that are collected through less invasive methods, has priority over the tissues. As blood serum contains the accumulation of metabolites from all body compartments, it seems difficult to find a specific blood metabolic pattern for a specific disease, however, metabolomics analytical methods following multivariate statistical analysis make it possible to effectively discriminate different groups as many studies were performed in this field and several potential biomarkers have been proposed for diagnostic or prognostic purposes based on omics studies including genomics, proteomics, and metabolomics (16). In this study, the metabolomics approach based on the NMR technique was utilized to find potential markers for discriminating malignant and benign thyroid nodules and to compare them with healthy subjects. The differential metabolites were also used to further understand the biochemical routes, which mostly involved in the thyroid cancer progression. Blood serum metabolic patterns demonstrated significant discrimination of patients with PTC as the malignant thyroid lesion from MNG as the benign lesion and from healthy controls, which indicated that metabolomics methods could be effectively used for the differentiation of disease conditions. Due to the fundamental role of the thyroid gland in regulating energy metabolism of the body, both malignant and benign thyroid lesions exert numerous variations in carbohydrates, lipids, and amino acids metabolism. From these variations, carnitine and acetylcarnitine were observed, the increment of them and their derivatives were widely reported in various tumors including oral, hepatic, and pancreatic cancers (17, 18). We also demonstrated higher amounts of carnitine and acetylcarnitine in MNG group but PTC samples did not show significant alterations of these metabolites. However, we found decreased amounts of acetylcarnitine in PTC samples compared to MNG, which was in accordance with a previous report about serum carnitines content in thyroid tumors (19). Altered acetylcarnitine in cancer cells is a sign of fatty acids beta-oxidation in mitochondria for providing energy. Among the main alterations in cancer metabolism, was the altered lactate level, which was elevated in both malignant and benign groups and is consistent with other studies. Alterations in lactate, alanine, and amino acids like asparagine, aspartate, glutamine, valine, and methionine, which play roles in gluconeogenesis indicate deregulation in energy metabolism. Cancer cells require large amounts of energy for the proliferation and biosynthesis of essential compounds which mainly take place through elevation of glucose and glutamine, lactate production, and biosynthesis of lipids. For the production of energy even in the presence of oxygen, tumor cells perform aerobic glycolysis, known as the Warburg effect, conducing to lactate, ATP, and NADPH generation in the cytosol, which is essential for cancer cell growth (20). In this study, alterations in citrulline and asparagine level were observed that were in accordance with some previous results (21). Citrulline is an amino acid having a role in the urea cycle and can act as a source for the feeding of the Krebs cycle (22). Variations of amino acids usually occur in tumor cells. In the MNG group, increased amounts of methionine, glutamine, citrulline, alanine, isoleucine, valine, and tryptophan were observed. There was also an increase in homocysteine, asparagine, aspartate, and tryptophan concentrations in PTC patients. Amino acid changes in different cancer studies do not lead to exactly the same results and differences in increase or decrease of these metabolite levels are impressed by various biological or technical factors. Free amino acid alterations may exhibit tumor-induced metabolism in patients with malignant and also benign lesions. In the study of Gu *et al.* significantly decreased amounts of aspartate, glutamate, proline, glycine, and totally non-essential amino acids were observed in the plasma of thyroid cancer patients. Significantly increased amounts of threonine, arginine, and essential amino acids were also reported besides methionine, leucine, tyrosine, and lysine. They showed that patients with thyroid cancer had much higher amounts of methionine, leucine, tyrosine, and lysine (23). However, in the recent study of Xu *et al.* increment of homocysteine, methionine, glutamine, aspartate, beta-alanine, valine, isoleucine, tryptophan, and hypotaurine was observed in PTC and benign lesions, which was in accordance with our results (24). Alterations in citrate content which is a Krebs cycle metabolite was also observed. Homoserine is the other metabolite with decreased amounts in PTC compared to MNG. Homoserine acts as an intermediate for the biosynthesis of methionine, isoleucine, and threonine. We observed increased amounts of scyllo-inositol, myo-inositol, and hypotaurine, which are related to osmoregulation and tumor volume alterations. Taurine, scyllo- and myo-inositol were altered in most of the previous metabolomic studies of thyroid carcinoma, which is in accordance with these results (2, 25-27). The other altered metabolite was choline, which besides its role in membrane phospholipids metabolism acts as a marker of cell growth (28). Choline is the precursor of glycerophospholipids, which makes the main part of biological membranes. Some phosphocholines aid in cancer cell growth by activation of some G-coupled receptors on cell surfaces (29). In almost all of the previous metabolomics studies of thyroid cancer, the increase of choline content and its derivatives including phosphocholine and glycerophosphocholine was reported in malignant thyroid lesions, which is consistent with our results (8). In our study, variations of ketone bodies and related compounds including acetone, acetate, and acetamide were observed that are related to lipid metabolism. Ketone bodies mainly produced from fatty acids β-oxidation, exhibit different patterns of alteration in cancer studies. Acetone and 3-hydroxybutyric acid were previously reported in thyroid lesions (19, 25). The other metabolite that increased in the MNG group was glutathione, but its alteration was not significant in PTC samples in our study. This metabolite is considered an index of tumor cells. Increased glutathione levels were observed in many studies related to carcinomas including thyroid cancer, which was in accordance with our results (2, 30, 31). The increment of GSH takes place following the increased metabolism of methionine and catabolism of glutamine and glutamate. GSH is considered an antioxidant and anti-ROS agent that performs this function here for the benefit of the cancer cells (32). Metabolite sets enrichment analysis (MSEA) was performed to seek the most significant biochemical pathways involved in PTC pathogenesis. As can be seen in Figure 3, the results of the pathway enrichment analysis showed that aspartate metabolism is the most important involved pathway in malignant thyroid lesions. Urea cycle and ammonia recycling were also from the significant pathways in the progression of PTC. The urea cycle is essential in converting excess nitrogen of ammonia and aspartate to urea to reduce its high toxicity. Somatic silencing of the ASS1 enzyme in the urea cycle was exhibited in many cancers and tumor tissues, and it was reported that loss of the ASS1 is associated with poor outcomes (22). Ammonia recycling is also important in cancer cell survival. In tumor cells with depleted glucose content, glutamine provides ammonia, triggering autophagy, which means these cells become more dependent on glutamine via glutaminase and glutamate dehydrogenase enzymes to produce α-ketoglutarate and provide sufficient energy for cancer cell survival by producing ATP and synthesis of amino acids, lipids and, nucleotides in the TCA cycle (33). It was also demonstrated that many cancer cells survive without glutamine and only use ammonia as a nitrogen source (34). It was, therefore, revealed that nitrogen and not the carbon skeleton underlies the importance of glutaminolysis for the proliferation of cancer cells (35). These important pathways are demonstrated in Figure 4.

## Conclusion

Thyroid carcinomas comprise the fastest rising incidence of cancer in the last decade for unknown reasons. Currently, diagnosis of thyroid tumors is performed by the FNAB method, which still holds some challenges and limitations, mostly in discriminating between malignant and benign lesions, therefore the development of molecular markers to distinguish between these lesion types are in progress. The blood metabolic signature has been introduced as an index for the study of various cancers in recent years and increasing metabolomics studies have been executed to search for markers of diagnosis, prognosis, and therapy. By the study of downstream metabolites, even small changes in genomic or proteomic levels could be multiple times amplified and quantitated. This study emphasized that the survey of blood serum by the NMR technique is a subtle and efficient method in order to find novel candidate markers and also for the study of the molecular basis of thyroid cancers. The metabolites discriminating between PTC and MNG included kynurenine, nicotinic acid, hippurate, citrate, xanthine, acetylcarnitine, glutamine, tryptophan, tyrosine, β-alanine, homoserine, and glutathione. The most important metabolites in PTC serum samples compared to normal subjects also included scyllo- and myo-inositol, tryptophan, propionate, lactate, homocysteine, 3-methyl glutarate, asparagine, aspartate, choline, and acetamide. Metabolism of amino acids, especially aspartate and methionine, and urea cycle were among the most significant biochemical pathways involved in the PTC pathogenesis. According to the increasing prevalence of thyroid cancers, the development of molecular markers could aid in better diagnosis and prevention of unnecessary surgeries. Validation of the results in larger cohorts is also of utmost importance for application of the results to the clinic.

## Conflicts of Interest

The authors declare that they have no conﬂicts of interest.
